# Is Pakistan's Response to Coronavirus (SARS-CoV-2) Adequate to Prevent an Outbreak?

**DOI:** 10.3389/fmed.2020.00158

**Published:** 2020-04-21

**Authors:** Bilal Javed, Abdullah Sarwer, Erik B. Soto, Zia-ur-Rehman Mashwani

**Affiliations:** ^1^Faculty of Sciences, PMAS-Arid Agriculture University, Rawalpindi, Pakistan; ^2^Roy & Diana Vagelos Laboratories, Department of Chemistry, University of Pennsylvania, Philadelphia, PA, United States; ^3^Nawaz Sharif Medical College, University of Gujrat, Gujrat, Pakistan; ^4^Allama Iqbal Memorial Teaching Hospital, Sialkot, Pakistan; ^5^Graduate School of Public Health, University of Pittsburgh, Pittsburgh, PA, United States

**Keywords:** novel coronavirus, SARS-CoV-2, COVID-2019 epidemic, pandemic, Pakistan

## Introduction

After the dreadful outbreak in Wuhan, China and scientific evidence of its human-to-human transmission, in an effort to stem the virus' reach and spread and to try to contain it at the source, governments across the world—most notably the United States—began putting in place and enforcing travel restrictions to and from China (1). However, because it was a new virus with little known about it and because there was a huge global dearth in the availability of screening and testing equipment, the disease spread rapidly across the world (2). In fact, it spread so rapidly that by 26th February 2020, the number of new infections outside of China had increased 13-fold when compared to the number of new infections inside of China. Additionally, the number of countries infected with COVID-19 had tripled. On 11th March 2020 WHO declared that COVID-19 could be categorized as a Pandemic. On 27 February 2020, Pakistan reported its first two patients of COVID-19 (3). The first two cases were from individuals who had recently traveled back to Pakistan from Iran (4).

**Figure 1:**
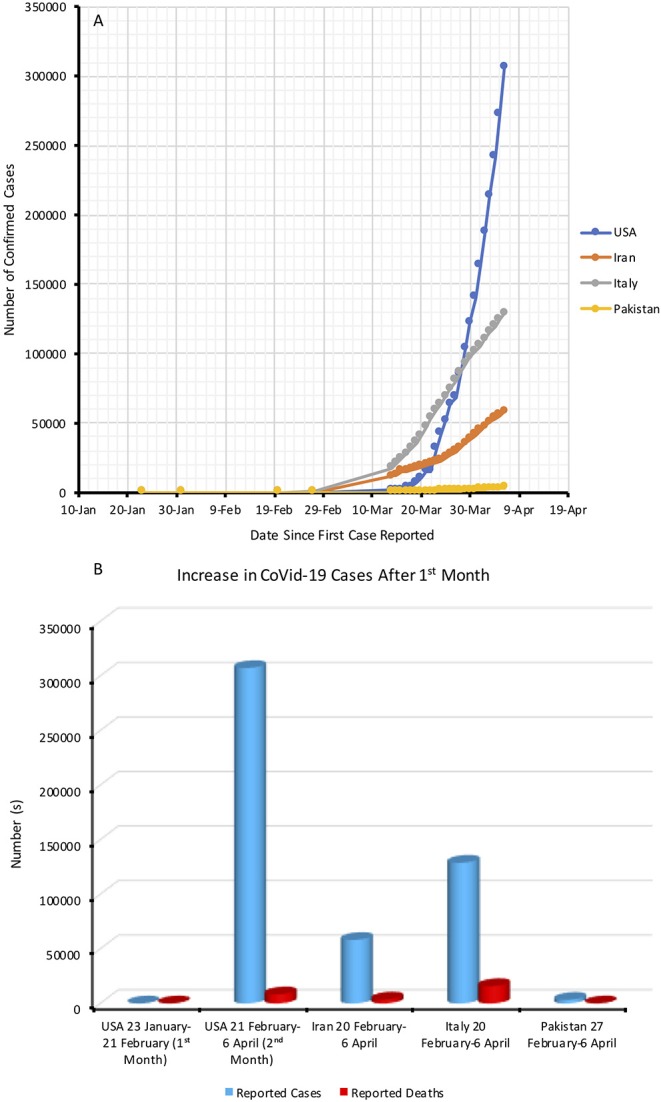
**(A)** Day-by-day increase in the number of COVID-19 cases since its first outbreak **(B)** Month-by-month increase in the transmission and death [Note: Data to plot graph is collected from WHO Coronavirus disease (COVID-19) daily situation reports].

As of 7 March 2020, the number of confirmed COVID-19 cases globally had surpassed 100,000, with 3,073 of them ending in death. Of these, 80,813 cases were from China while 21,110 confirmed cases with 413 deaths were from outside of China. On that date, the number of cases in Iran was 4,747 with 121 of them ending in death (5). Pakistan reported 7 cases with no mortalities. By the 7th April 2020, WHO reported 1,214,466 confirmed cases and 67,767 deaths across 211 countries, areas, or territories (4) (Figure 1).

## Pakistan's Government Response to COVID-19

China, an epicenter of COVID-19, is located northeast of Pakistan. Additionally, Pakistan shares its southwest border with Iran where the number of cases and deaths are increasing exponentially. The geographical locations of extremely severe outbreaks in two countries that border Pakistan (China and Iran), in addition to the declaration of COVID-19 as a pandemic by WHO, forced Pakistan's government to take drastic, severe, and quick actions to stop the further transmission of the virus in the country (6). Notwithstanding this, the current trade agreements with China and the politico-religious relationship with Iran has resulted in the influx of infected individuals from these two regional epicenters of the virus. To curtail further transmission, as a first-line response Pakistan closed the border with China and put very strict screening methods at the Pakistani-Iranian border (2). Additionally, in coordination with the civil aviation authority, the government of Pakistan enforced the screening of passengers before they would be allowed to enter the country (7). However, in the earlier days of the pandemic, Pakistan lacked the ability to diagnose COVID-19 directly and therefore the country had to rely on China, Japan, and the Netherlands to test their samples. This resulted in a crucial time lag and caused delays in the government's ability to adequately respond to the virus. Fortunately, the government did eventually receive diagnostic kits from China and primers from Japan to be able to test samples on their own (5). WHO also designated seven hospitals nationwide to test suspected COVID-19 patients (8). Pakistan's federal government, with the collaboration of the Ministry of Health, devised a plan which was called, “*The National Action Plan for The Corona Virus Disease (COVID-19) Pakistan”* (6). The purpose of this plan was to devise policies and a template to help provincial governments and states across Pakistan with a guide for them to develop methods and strategies to best deal with the COVID-19 outbreak. Using this guidance, provincial governments established quarantine centers at Lahore and Karachi's (two of the country's biggest cities) exposition centers with the help of the armed forces of Pakistan (9). A newly constructed apartment building in the city of Sukkur was also designated as a quarantine camp by the government with 2,000 beds (10). Additionally, another quarantine center was also established in the city of Taftan along the Pakistani-Iranian border to help identify and quarantine individuals returning to Pakistan after spending time in Iran (11). A very modern quarantine center was established in Islamabad with 300 beds. The government also ordered the closure of all hotels and, by invoking special powers, designated some of them as quarantine centers. Apart from these containment facilities, the government also established isolation wards in many hospitals (12). The Ministry of Health also managed to provide crucial supplies to the fight of this disease such as face masks, gloves, and protective suits to protect the paramedical staff and doctors at the frontlines of this pandemic. Hospitals started primarily dealing with crucial emergencies and COVID-19 patients (5). Telephone helplines were established by the provincial governments for people to inquire about COVID-19 related healthcare issues. They also used this platform to let callers know that they should stay at home if they start experiencing any symptoms of the virus. Campaigns were launched throughout the nation's traditional media and social media outlets to increase awareness among the general public about proper hand sanitization techniques and the importance of social distancing to break the chain of transmission. The government distributed alcohol-based sanitizers to people in need and the manufacturing of disinfectant walkthrough gates also began, with some installed at the entrance of some food markets (6).

However, even with all of these efforts, major lapses existed at every step. Issues include the inconsistent implementation of immigration policies dealing with the influx of people from the borders and airports (7) to the lack of crucial protective suits and other supplies in hospitals (2). Consequently, the lack of facilities, poor infrastructure, and inconsistent implementation of government policies resulted in the rapid and continuous spread of COVID-19 throughout the country (10, 11, 13).

Hospital staff protested working without adequate protective supplies (14). What is more, quarantine centers were perceived as under-performing in serving to isolate infected individuals from the healthy populace. The one-room one-person policy was badly neglected along with the lack of clean bathrooms and drinking water. Five people were reported to be living in one single containment camp (11). Meanwhile, the government planned to shift COVID-19 infected individuals directly to Multan and Faisalabad (large Pakistani urban centers) after changing some of those cities' public university dormitories to quarantine centers (13). Hoarding and black-market selling of protective goods to the public resulted in a lack of protective supplies for the country's healthcare practitioners. To mitigate this issue, the National Disaster Management Authority (NDMA) and the Drug Regulation Authority (DRA) stepped in to help the government prevent hoarding and the black-market trade of protective supplies (5).

Fear of a national economic downturn to an already troubled economy coupled with the fear of a decline in jobs and in the ability of the average citizen to earn and provide for their families further hampered the ability of the government to lockdown cities and markets to curtail the transmission of the pathogen, as ordinary citizens ignored governmental calls and ordinances urging people to stay at home (15). The package worth 900 billion Pakistani rupees ($5.66 billion) was approved in a Cabinet meeting to support low-income groups, particularly labor, and to improve health care facilities in public hospitals (16). However, the shortcomings and challenges mentioned above maintained an ineffective containment of the COVID-19 outbreak in Pakistan.

## Public Response to COVID-19

The initial response of the public to the emerging threat of COVID-19 was that of a generally reported apathy and indifference. Lack of public awareness was commonplace throughout the country and mass prayer events continued even as alarms were set off as to how such public activities could exacerbate the spreading of the pathogen (17). The spread of misinformation, of fears, rumors, and false facts was initially rife throughout social media. The price of common utilities quickly grew in the face of regional countries severing international trade in an attempt to hamper the spread of the virus, in addition to the black-market selling of essential goods and the public hoarding of many products (18). However, the regional price control authorities started monitoring commodity prices on the instruction of the federal government (18).

Individual incidences came to light, such as how a person traveling from Spain managed to evade the airport screening booth after testing positive which resulted in the transmitting of the disease to his family and community (19). What is more, some people broke their quarantine at the Sukkur camp and left their rooms, coming into direct contact with others and further spreading the disease (10). The indifference and non-cooperative attitude displayed by the general public further fueled the rapid transmission of the disease across the country.

## Current Situation in Pakistan and Preliminary Clinical and Scientific Investigation

The Ministry of Health on 27 February 2020 reported the first two COVID-19 cases in the city of Karachi by individuals who had traveled to Iran and then returned to Pakistan (6). In less than a month from then, WHO reported 784 (~392-fold increase) cases and five mortalities. Conversely, the number of cases in the US jumped to 15 in the first month after they reported their first infection in late January. Italy (59,138) and Iran (21,638) also reported a surge in transmission and deaths (Figure 1A). A comparison of WHO's reported day-by-day data from Pakistan, the US, Italy, and Iran shows (Figure 1B) that Pakistan could be the next country to see an exponential rise in COVID-19 transmission and death (4).

Pakistan's scientific community is working alongside scientists, health professionals, and various governments from across the world to find a cure or different ways to manage this condition. Pakistan's biological community volunteered to help health professionals perform diagnostic tests such as PCR. A scientific team from the National University of Science and Technology and the University of Punjab separately developed low cost diagnostic kits that will be manufactured en masse within Pakistan, saving time and money (5). Dr. Tahir Shamsi (20), head of the National Institute of Blood Diseases (NIBD) in Karachi, has advocated for the use of a medical technique known as passive immunization, that involves the administration of antibodies from a COVID-19 cured patient to a non-immune individual and is used when the risk of infection is high, the time for the human body to generate an immune response is low, and no vaccine is available (21).

However, the current pandemic which stemmed from China and has resulted in the large-scale illness and deaths of both people in Iran and Italy and across the globe, should compel the Pakistani government to take further drastic and timely measures. The current situation requires the politicians, health professionals, scientists, and the general community to band together in taking steps to fight this pandemic. It is highly regarded that the US, Italy, and Iran have a better health care system than Pakistan (5). Notwithstanding this, these countries have failed drastically to contain the virus largely due to inconsistent policies and late decisions and actions. Their failures should prompt the Pakistani government to make timely decisions and enforce them to prevent further transmission of the disease. Otherwise, with the limited available health care facilities and poor infrastructure in place, the outbreak in Pakistan may soon mirror the situation in Iran and Italy.

## Author Contributions

BJ devised the study, designed, collected, analyzed the data, and wrote the first draft. AS, ES, and ZM edited and revised the subsequent drafts. The authors reviewed and endorsed the final submission.

## Conflict of Interest

The authors declare that the research was conducted in the absence of any commercial or financial relationships that could be construed as a potential conflict of interest.
